# Economic analysis of participation in physical activity in England: implications for health policy

**DOI:** 10.1186/s12966-014-0117-9

**Published:** 2014-09-14

**Authors:** Nana Kwame Anokye, Subhash Pokhrel, Julia Fox-Rushby

**Affiliations:** Health Economics Research Group (HERG), Brunel University London, Uxbridge, Middlesex, UB8 3PH, London, UK

**Keywords:** Physical activity, Prices, Time, Money, Participation, Demand

## Abstract

**Background:**

Changing the relative price of (in) activity is an important tool for health policies. Nonetheless, to date, analyses of correlates of physical activity (PA) have excluded the notion of price. Using the first nationwide dataset on prices of PA for England, we explore for the first time how money and time prices are associated with PA (in general) and specific activities.

**Methods:**

A nationally representative telephone follow-up survey to Health Survey for England (HSE) 2008 was undertaken in 2010. The sample covered individuals who reported to have undertaken some PA in the HSE 2008. Questions focussed on: ex-post money and time prices; type and quantity of PA; perceived benefits of PA and socio-economic details. Count regression models (all activities together, and swimming, workout, walking separately) were fitted to investigate the variation in quantity of PA.

**Results:**

Of 1683 respondents, 83% participated in PA (one or more activities), and spent an average of £2.40 per occasion of participation in PA and 23 minutes travelling. Participation in PA was negatively associated with money prices per occasion (i.e. family member/child care fees, parking fees, and facility charges) and travel time price. Participation in PA was more sensitive to travel time price than money price. Among the specific activities, the money price effect was highest for swimming with a 10% higher price associated with 29% fewer occasions of swimming; followed by workout (3% fewer occasions) and walking (2% fewer occasions). Only swimming and workout were sensitive to travel time price. People who felt doing PA could help them ‘get outdoors’, ‘have fun’, or ‘lose weight’ were likely to do more PA.

**Conclusions:**

Two main policy implications emerge from the findings. First, the results support the notion that positive financial incentives, e.g. subsidising price of participation, could generally lead to an increase in quantity of PA among those already exercising. Second, such policies could lead to desired policy goals if implemented at an individual activity level (e.g. 50% subsidy on swimming entrance charges) rather than a blanket implementation (e.g. subsidising average entrance charges across all activities by 50%).

## Background

Insufficient physical activity is associated with increased risks of developing over 20 health conditions including cardiovascular disease, cancer, obesity and diabetes. The global public health agenda aims to increase participation in physical activity (PA).

Changing the relative price of (in)activity is an important tool for public policy and public health. Despite this it is noticeable that studies of the determinants of demand for PA have excluded the notion of price [1,2], largely because the majority of literature on determinants has not included economists [3]. Priced-based policies could include: use of subsidies e.g. to the leisure industry to increase provision of accessible services; use of tax incentives (e.g. reductions on tax of sport equipment, reduced prices or vouchers for sport sessions e.g. swimming, increasing goods/services associated with inactivity such as petrol, road use). Each of these policies has the potential to increase activity not only among the inactive but also among those who are active but do not participate sufficiently often or with sufficient intensity to gain the health benefits. The latter is the focus of this present study.

Scoping reviews have found that very few studies have examined the impact of economic instruments including prices to promote PA and consequently that clear policy recommendations cannot yet be made [4]. A more recent systematic scoping review [5] also found no primary studies of price promotions or supply side impacts and few studies of the impact of tax or transfer payments. The exceptions included impacts of congestion charging, taxes on beer and cigarettes, employer-sponsored transfer payments for use health clubs or travel to work using public transport, and tax credits to parents for enrolment of their children in organised PA programmes [6,7]. These findings contrast with the quantity of such evidence found for diet/food [4,8] – the main contributor along with inactivity to increasing obesity.

The limited economic literature on PA has focussed on non-price determinants (e.g. education, income, social class) [9,10] although some have noted the importance of time and money prices in understanding demand for PA [11–13]. The role of prices in understanding participation in PA is not straightforward. Unlike other commodities, PA is not just a consumption good for which a regular market price is paid. To undertake PA, different goods and services as well as time may be required; and hence variety of prices includes entrance charges; classes fees; membership fees; and parking charges [14–16]. For example, per occasion of swimming, equipment (e.g. goggles); family/child care; facility (e.g. sports complex); travelling time, and parking bay may be needed.

The few studies, mainly from the US, that have explored the effects of price have done so partially, with attempts limited to: assessing the impact of time price [15,17,18] or money price (e.g. entrance or membership fees) alone [19–21] or using proxy measures for time price through (education and income) and a limited number of area based prices of: bowling, tennis balls, and gas, and bus fare [22]. While Anokye et al. [16] had a broader specification of money prices and specified time price as travel time, the sample was limited to staff and students at an university.

The findings of existing studies are mixed. Brown and Roberts [17] found a mixed impact for opportunity cost of time, with the results moderated by type of model and gender. McInnes and Shinogle [22], and Humphreys and Ruseski [15,18,23] found a mixed effect depending on various specifications of PA. Regarding money price, Tai et al. [20] found no effect for entrance charges while Coalter [19] found mixed effect and PwC [21] a negative effect. McInnes and Shinogle [22] found no effect for area level prices of tennis, and bowling balls, but a negative effect for gas prices and positive effect for bus fare. Anokye et al. [16] showed demand for PA was negatively associated with travel time price per occasion and money price per occasion but positively correlated with monthly price.

Humphreys and Ruseski [15] US study, suggests that relationship between price and PA might differ depending on specific activities and points to the need for further research. However, to date, this has not been explored elsewhere. Activity specific analysis is important because it could allow policy to target specific activities.

The aim of this study is to show for the first time how the participation in PA is associated with time and money prices using the first nationwide English dataset on prices of PA. We address the gaps in the literature by demonstrating whether and (how) different money prices and travel time price relate to participation in PA (in general- one or more activities) and individual activities. Using the UK as a case study provides a new perspective to improve our understanding of the limited evidence base, which to date, is US dominated. This is important given the varying health systems in both countries.

## Methods

### Theoretical framework

A utility (satisfaction) maximisation approach is used to explain why some individuals do more PA than others. Individuals derive utility (now and in the future) from consumption of PA and other goods, subject to budget and time constraints. Utility is maximised when the marginal rate of substitution (i.e. the rate at which individuals are willing to trade off other goods for PA) equals the ratio of time and money prices of PA and other goods. This is equal to the ratio of marginal utilities that individuals’ derive from the consumption of PA and other goods. Hence a change in time and money price (all other things being equal) could change the quantity demanded of PA. When the price(s) of PA falls (all other things being equal), the equi-marginal principle is unbalanced as the individual obtains greater utility from an extra unit of PA than from other goods. To maintain the original level of utility, the individual will consume more of PA. It is therefore hypothesised that prices of PA correlate negatively with the quantity of PA, holding other factors constant. To date, the application of this well-established general theory and the strength of this theory in this area could, however, be considered weak as there is insufficient empirical evidence in this area [9,10] and the limited evidence has presented mixed findings[15–18,22,23]. This study therefore presents the basis for hypothesis generating.

### Data

Data came from Economics of Physical Activity Survey (EPAS). EPAS is a follow-up survey to the Health Survey for England (HSE) 2008 and draws a representative sample of 1683 adult residents in England who, at the time of HSE 2008, had participated in PA (one or more activities) in the preceding four weeks and had indicated they were willing to be contacted again. A two-stage stratified random sampling approach was used (based on households and then individuals within households). EPAS was undertaken in 2010 by telephone and covered the following topics: indicators of PA behaviour; money and travel time prices of participation; and socio-economic, psychological and demographic details. Respondents were screened into this follow-up only if they had participated in PA in past 4 weeks.

Indicators of PA behaviour were captured as frequency, duration and intensity of participation in various activities (e.g. swimming, cycling, working out using an exercise machine/weight training either in a gym/home, team sports, running/jogging) during the four weeks prior to survey date. To reduce respondent burden and work within time constraints, if the respondent indicated participation in more than three activities in the past 4 weeks, three of those activities were randomly selected for the further questioning.

Price data was collected as ex-post price (i.e. amount paid) and not ex-ante (facing price). Respondents were asked about: membership fees; joining fees; entrance charge; class fees; hiring of court/pitch; buying/hiring of equipment; buying/hiring of shoes or clothing; maintenance of equipment or clothing; buying of food or drink consumed whilst doing activity; nutritional supplements; child or family member care. To ensure that all prices relating to an activity were captured, a ‘catch all’ prices question was asked at the end of the interview. This allowed respondents to report anything that they had forgotten to mention earlier.

For all money prices, data was collected for price paid per occasion of PA or monthly price (a ‘one-off’ price paid each month irrespective of the number of occasions they undertook PA). These prices are mutually exclusive. Travel time price was captured per occasion and measured as the travel time (in minutes) to do an occasion of PA.

Data was also collected on socio-economic, psychological and demographic variables that had been shown to correlate with PA (hereafter referred to as control variables). These covered: income; ethnicity; gender, age, employment status; education; smoking status; marital status; number of adults; number of children; region; access to vehicle drinking status; past BMI; urbanisation; previous region of residence; past health status; and perceived benefits of PA. Notably, a number of these including health status, BMI, and area-based factors were based on HSE 2008 data. Perceived benefits of PA were measured using two scores from 5 benefits. These benefits were: ‘to have fun’; ‘to get outdoors’; ‘to get mentally alert’; ‘to reduce weight’; ‘to improve/maintain health’. The first score reflects how much a person thinks PA could help him/her achieve the 5 items rated 1 to 5 (1 = could not help you at all; 2; 3; 4; 5 = could help you a great deal). The second reflects the respondent’s view of the importance of the benefit (RIPB) from the 5 items to themselves. The ratings ranged from 1 to 5 (1 = not at all important; 2; 3; 4; 5 = very important).

### Data analyses

#### Missing observations and their replacement

There were two types of missing data. First, data could be missing because the respondent reported more than three activities. This type of missing data applied only to level of PA participation and prices of those activities that were not randomly selected. A mean-based imputation method was used where data were missing at random. The second type of missing data occurred due to non-response and the mechanisms under which the missing data occurred (i.e. missing completely at random or not) was examined using chi square and Fischer’s exact tests to check the association between the indicator of PA and dummy variables representing item non-responses. If the pattern of missing data did not occur completely at random (i.e. PA is significantly associated with item non-response), a regression-based imputation method was used to replace missing values of continuous variables and a dummy variable specifying item-non response added. If the pattern of ‘missingness’ occurred completely at random, a mean-based imputation method was used to replace missing values. For the categorical variables, irrespective of the mechanism under which ‘missingness’ occurred, missing values were treated as separate categories in order to observe potentially informative non-responses.

#### Regression models

To allow combined activities and activity specific analyses, the dependent variables were specified as the number of occasions on which PA were undertaken for: (a) all activities combined; (b) swimming, (c) working out using an exercise machine or weight training, either in a gym or at home; and (d) walking. Resource constraints precluded ‘activity specific’ analysis for each individual activity identified in this study and hence the most popular activities and those relevant to the UK policy context were selected.

The main independent variables were specified as travel time and money prices (prices per occasion and monthly price per activity). Money prices were selected into the regression on the basis of a multivariate analysis that investigated whether they were likely to be statistically significant. Other independent variables included the control variables.

Count regression models were fitted for combined, and activity specific analysis. The negbin variant of count models was used as the estimated alpha parameters were greater than zero and highly significant (p < 0.001); and, the dependent variables had no zero observations. Sample selection bias was examined using Heckman selection models to check whether the sample of participants in PA is representative of sports participants in the population.

All models were subject to standard diagnostic tests. Marginal effects and elasticities, estimated at sample mean values of independent variables, were computed for each variable. Elasticities were calculated for continuous variables and marginal effects for categorical variables. For the price variables, the elasticity estimates’ indicate the price elasticity of demand for physical activity, which shows the responsiveness of quantity of PA to variation in prices. An elasticity estimate >1 (<1), price elastic (inelastic), shows that quantity of PA responds more than proportionately to variations in price (vice versa). The threshold for statistical significance was set at ≤10% in all analyses because of the exploratory nature of the study. All analyses were undertaken using Stata version 10.

Categorical independent variables including perceived benefits, and other explanatory variables were collapsed into a binary variable if found not to have sufficient observations in categories to allow comparison across those categories. Based on previous research, we hypothesise that perceived benefits (high), income, educational qualification, and past health status would be positively correlated with PA [10,16,24,25]. Age, and being female, full-time employed, a smoker or non-drinker were expected to have a negative relationship with PA [24,26]. The expected correlation for ethnicity (white), marital status & cohabitation (married), past BMI, region of residence (north east), urbanisation (urban), access to vehicle (yes), number of children, and adults in the household is not clear as past research yielded mixed findings [24,26].

## Results

### Description of sample

The majority of the sample (83%, n = 1393) had done PA in the past four weeks whilst 17% (n = 293) had not. The remaining results are presented only for those who participated in PA. On average, people participated in 2 different activities. 89% did a maximum 3 and one person participated in 7 different activities. 72 different activities were identified with the most popular including workout, swimming, walking, running, and cycling whilst the least common included windsurfing, water polo, skipping, and surfing. On average, people did PA on 21 occasions in the 4 weeks prior to the survey. Those who undertook swimming, workout, and walking did so on 5, 9, and 20 occasions respectively (on average).

The sample was predominately White (92%) and aged between 17 and 54 years (60%). Of the sample, 51% were female. Most were married and living with their partners (54%), and reported favourable past health status (84%). Few were defined as obese (19%) or smokers (7%), though the majority were ‘drinkers of alcohol’ (92%). Most participants were employed (61%), had an annual household income above £18,200 (58%), and educated with qualifications ranging from degree to NVQ1 (87%).

All 5 aspects of perceived benefits were scored 3 or greater, indicating that the respondents would perceive all these benefits arising from PA participation. ‘To improve or maintain your health’ was the most commonly perceived benefit (median (IQR):5(4, 5); score 3 to 5: 95%), and ‘to reduce weight’ the least (median (IQR): 4(4, 5); score 3 to 5: 75%). All 5 items listed as possible perceived benefits had a median score of 3 or more (score 3 to 5: 89%) for importance to them.

### Missing observations

Eleven percent of the sample reported more than three activities and had their missing data replaced for those activities that were not randomly selected. There was no missing data for the dependent variables. However, among the independent variables, income, ethnicity, prices, working hours, BMI, access to vehicle, and perceived benefits had missing observations. BMI had the highest number of missing observations (9%) while prices, ethnicity, and working hours had the lowest (>1%). All these data were found to be missing at random and were adjusted for in the analyses to ensure maximum use of data.

### Prices

Table 1 shows the distribution of the different money prices paid for participation in PA. Per occasion of participation in PA, the full sample spent £2.40 on average. 31% (n = 436) spent nothing. Prices paid per occasion or monthly were mostly for entrance charges, and membership fees respectively.

**Table 1:** **Descriptive statistics of money prices related to PA (2010 £ sterling)**

**Price**	**Mean(SD) of full sample**	**No. of positive values (no. of zeros)**	**Mean(SD) of partial sample** ^**a**^
*Membership fees*			
Price per occasion	0.01(0.23)	16(1377)	1.05(1.88)
Monthly price	23.96(112.85)	449(944)	74.35(189.25)
*Joining fees*			
Price per occasion	0.003(0.09)	54(1339)	0.07(0.44)
Monthly price	1.20(24.72)	111(1282)	15.04(86.73)
*Entrance charges*			
Price per occasion	0.54(4.69)	210(1183)	3.61(11.64)
Monthly price	1.21(11.77)	153(1240)	10.99(34.06)
*Classes fees*			
Price per occasion	0.39(1.65)	219(1174)	2.45(3.52)
Monthly price	1.75(13.50)	144(1249)	16.94(38.91)
*Court/pitch charges*			
Price per occasion	0.17(1.21)	66(1327)	3.54(4.37)
Monthly price	0.97(24.47)	67(1326)	20.24(110.61)
*Equipment hire/purchase*			
Price per occasion	0.32(4.92)	44(1349)	10.20(26.10)
Monthly price	2.18(17.08)	163(1230)	18.62(46.88)
*Apparel hire/purchase*			
Price per occasion	0.0001(0.01)	3(1390)	0.07(0.12)
Monthly price	1.74(9.29)	163(1230)	14.91(23.32)
*Repair of equipment/apparel			
Price per occasion	0.02(0.67)	25(1368)	1.02(5)
Monthly price	0.79(16.32)	104(1289)	10.62(59.10)
*Food/drinks purchase*			
Price per occasion	0.26(1.39)	228(1165)	1.57(3.13)
Monthly price	0.48(6.62)	162(1231)	4.16(19.07)
*Nutritional supplements purchase*			
Price per occasion	0.01(0.13)	92(1301)	0.14(0.47)
Monthly price	0.43(3.53)	135(1258)	4.43(10.58)
*Family/child care fees*			
Price per occasion	0.02(0.36)	72(1321)	0.31(1.58)
Monthly price	0.02(0.47)	72(1321)	0.37(2.05)
*Transport fare			
Price per occasion	0.14(2.80)	78(1315)	2.54(11.67)
Season ticket	0.08(1.62)	39(1354)	2.78(9.43)
*Extra expenses*			
Price per occasion	0.09(3.14)	44(1349)	2.77(17.62)
Monthly price	5.02(123.80)	16(1377)	437.13(1104.9)
*Parking fees*			
Price per occasion	0.003(0.07)	8(1385)	0.50(0.75)
Monthly price	0.01(0.24)	31(1362)	0.48(1.55)
*Facility charges*			
Price per occasion	0.0005(0.01)	71(1322)	0.01(0.05)
Monthly price	0.06(2.04)	35(1358)	2.22(12.84)
*Competition fees*			
Price per occasion	0.01(0.41)	5(1388)	3.81(6.38)
Monthly price	1.51(48.57)	21(1372)	99.88(392.29)
*Accommodation charges*			
Price per occasion	0.10(3.20)	11(1382)	12.99(35.18)
Monthly price	2.15(39.22)	42(1351)	71.45(217.12)
*Sports holiday*			
Price per occasion	0.01(0.34)	5(1388)	2.53(5.57)
Monthly price	10.03(253.53)	20(1373)	698.47(2050.11)
*Other travel expenses*			
Price per occasion	0.21(6.34)	12(1381)	24.13(66.71)
Monthly price	0.62(12.74)	23(1370)	37.56(93.88)

^a^This refers to observations with positive values for the respective prices.

*These prices were not entered in the main model as they were found not to be significant.

People spent an average of 23 minutes travelling per occasion of PA, with half spending up to 10 minutes and one person spending 12 hours. 29% (n = 406) spent no time travelling to do PA. Excluding those with no travelling time, increases the average to 33 minutes per occasion of PA Three prices were not entered in the ‘all activities combined regression model’ as they were found not to be significant in the analyses to select prices. These prices include: repair of equipment/apparel, transport fare.

### Regression models

Tables 2 and 3 show the estimates for all regression models. Models had good specification, satisfied appropriate statistical assumptions and additional statistically significant independent variables could only be found by chance. There was no evidence of collinearity as the average variable inflated factor for the variables was 1.5, and average tolerance levels were 0.7. The estimates of the regression models could be considered unbiased as no evidence of selection bias was found using different sets of exclusion criteria. The correlation between the error terms of the selection and outcome models was not statistically significant (p value = 0.110 to 0.620). Results are first presented for the ‘all PA activities combined’ and then for each ‘activity specific’ model. The correlation between demand and money prices, travel time price, and control variables are presented for each.

**Table 2:** **Estimates of ‘all PA activities combined’ regression model**

**Independent variables**	**No. of occasions of PA (in general)**	
	***Coef.*** ^***a***^	***ME /Elas’ty*** ^***b***^
**Prices**		
Classes fees (occasion price)	0.056***	0.424
Classes fees (monthly price)	0.007***	0.250
Facility charge (occasion price)	−4.755**	−0.045
Facility charge (monthly price)	−0.022*	−0.024
Family/child care fees (occasion price)	−0.129*	−0.040
Sports holiday (monthly price)	0.000*	0.031
Travel (monthly price)	0.004*	0.049
Parking fees (occasion price)	−0.799**	−0.045
Accommodation charges (occasion price)	0.016**	0.033
Travel time price	−0.003***	−1.150
**Control variables**		
***Perceived benefits***		
*To get outdoors*		
Did not feel that PA can help them get outdoors (omitted category)		
Felt that PA can help them get outdoors	0.293***	5.207
*To have fun*		
Did not feel that PA can help them have fun (omitted category)		
Felt that PA can help them have fun	0.206**	3.783
*To control or lose weight*		
Did not feel that PA can help them control/lose weight (omitted category)		
Felt that PA can help them control/lose weight	0.136**	2.600
***Annual income £ (Joint income-partners/single)***		
<10,400 (omitted category)		
10,400 to less than 18,200	−0.067	−1.305
18,200 to less than 28,600	0.126*	2.581
*28,600* to less than *46,800*	0.030	0.598
46,800 and above	0.064	1.288
***Educational qualification***		
National vocational qualification 4 or 5/degree or equivalent (omitted category)		
Higher education below degree	0.124	2.563
National vocational qualification 3/general certificate of education advanced level equivalent	0.030	0.583
National vocational qualification 2/general certificate of education ordinary level equivalent	0.013	0.256
National vocational qualification 1/certificate of secondary education level equivalent	−0.294*	−5.093
Foreign/other	−0.034	−0.669
No qualification	0.015	0.304
***Employment status & working hours***		
Fulltime (omitted category)		
Part-time	0.141**	2.900
Unemployed	0.145**	2.912
***Health status***		
Very good (omitted category)		
Good	−0.009	−0.168
Fair	0.017	0.346
Bad	0.305**	6.996
***Marital status & cohabitation***		
Married & living with partner (omitted category)		
Living with someone as part a couple	−0.035	−0.680
Living alone	0.165**	3.341
***Region of residence***		
North east (omitted category)		
North west	0.215**	4.600
Yorkshire and the Humber	0.148	3.120
East midlands	0.149	3.116
West midlands	0.212*	4.557
East of England	0.102	2.090
London	0.193	4.109
South east coast	0.230*	4.957
South central	0.298**	6.688
South west	0.223*	4.803
*No. of observations*	1393	
*Constant*	2.163***	
*Linktest*	*p* = 0.808	
*Mcfadden Pseudo R squared*	0.006	

^a^Coefficient; ^b^Marginal Effects/Price Elasticity of Demand; Significance level of 1%(***), 5%(**), 10%(*).

**Table 3:** **Estimates of activity specific models regression models**

**Independent variables**	**Swimming**		**Workout**		**Walking**	
	***Coef*** ^***a***^ ***.***	***ME /Elas’ty*** ^***b***^	***Coef*** ^***a***^ ***.***	***ME /Elas’ty*** ^***b***^	***Coef*** ^***a***^ ***.***	***ME /Elas’ty*** ^***b***^
**Prices**						
Membership fees (monthly price)	0.002***	0.334				
Joining fees (monthly price)	−0.007*	−0.052	−0.016**	−0.083		
Entrance charges (monthly price)	1.724**	2.840				
Entrance charges (occasion price)	−1.714**	−2.864	−0.180***	−0.285		
Purchase/hiring of equipment (monthly price)	0.022*	0.024	0.004**	0.081		
Accommodation charges (monthly price)	0.001***	0.021				
Food/drinks (occasion price)			0.047**	0.062		
Classes fees (occasion price)			−0.044**	−0.059		
Repairs of equipment/apparel (monthly price)			−0.088**	−0.080		
Nutritional supplements (occasion price)			0.203*	0.035		
Family member/child care (monthly price)			0.203*	0.024		
Classes fees (monthly price)					0.008*	0.305
Walking parks (occasion price)					−0.759**	−0.190
Travel time price	−0.005***	−0.543	−0.003**	−0.361		
**Control variables**						
***Age (in years)***						
17-24 (omitted category)						
25-34			−0.206*	−1.751		
35-44			−0.181*	−1.581		
45-54			−0.159	−1.385		
55-64			−0.075	−0.673		
65-74			−0.079	−0.698		
75+			0.316**	3.365		
***Annual income (£)***						
>10,400 (omitted category)						
10,400 to less than 18,200	−0.260**	−1.054				
18,200 to less than 28,600	0.038	0.172				
28,600 to less than 46,800	−0.008	−0.037				
46,800 and above	0.012	0.052				
***Educational qualification***						
National vocational qualification 4 or 5/degree or equivalent (omitted category)						
Higher education below degree					0.060	1.165
National vocational qualification 3/general certificate of education advanced level equivalent					0.034	0.661
National vocational qualification 2/general certificate of education ordinary level equivalent					0.082	1.614
National vocational qualification 1/certificate of secondary education level equivalent					−0.537***	−8.047
Foreign/other					−0.589	−8.581
No qualification					0.050	0.976
***Employment status & working hours***						
Fulltime (omitted category)						
Part-time			−0.059	−0.526		
Unemployed			−0.139*	−1.236		
***Drinking status***						
Almost every day (omitted category)						
Five or six days a week	−0.081	−0.347			−0.462*	−7.274
Three or four days a week	−0.194	−0.819			0.039	0.760
Once or twice a week	−0.066	−0.289			−0.113	−2.113
Once or twice a month	−0.199	−0.824			0.064	1.249
Once every couple of months	−0.381*	−1.433			−0.207	−3.612
Once or twice a year	−0.033	−0.146			−0.138	−2.474
Not at all in the last 12 months/non-drinker	−0.023	−0.101			−0.273	−4.668
***Number of children in household***						
0 (omitted category)						
1	−0.171*	−0.718				
2	−0.466***	−1.793				
3 and above	−0.080	−0.344				
***Urbanisation***						
Urban (omitted category)						
Town	−0.212*	−0.866				
Village/hamlet	−0.167	−0.697				
***Marital status & cohabitation***						
Married & living with partner (omitted category)						
Living with someone as part a couple	−0.213*	−0.871				
Living alone	0.196	0.900				
**Access to vehicle**						
Yes (omitted category)						
No			0.182**	1.785	0.332**	7.278
*No. of observations*	362		534		231	
*Constant*	1.804***		2.437***		2.969***	
*Linktest*	*p* = 0.404		p = 0.966		p = 0.784	
*Pseudo R squared*	0.071		0.022		0.015	

^a^Coefficient; ^b^Marginal Effects/Price of Elasticity of Demand; Significance level of 1%(***), 5%(**), 10%(*).

### ‘All PA activities combined’ models

#### Money prices

Table 2 shows that the number of occasions on which PA were undertaken was negatively related with occasion price for facility charges (Price Elasticity of Demand (PED) = −0.045) meaning that a 10% higher charge is associated with less than 1% fewer occasions participating in PA (all things being equal). Other money prices that are negatively associated with the number of occasions of participating in PA were; occasion prices for family member/child care (PED = −0.040), parking fees (PED = −0.045) and monthly price for facility use (PED = −0.024). A positive correlation was observed for monthly prices for: class fees (PED = 0.250), sports holidays (PED = 0.031), other travel (PED = 0.049), and occasion prices for class fees (PED = 0.424) and accommodation (PED = 0.033). Demand was money price inelastic (for all prices), irrespective of direction of correlation.

#### Travel time price

The relationship between travel time price and number of occasions on which PA was undertaken was found to be negative and demand was travel time price elastic indicating that at the mean travel time price of 23 minutes, a 10% higher travel time price is associated with about 12% fewer occasions participating in PA (Table 2).

#### Control variables

Perceived benefits including ‘to get outdoors’; ‘to have fun’; and ‘to control or lose weight’ were positively correlated with the number of occasions on which PA was undertaken (Table 2). ‘To get outdoors’ was the most important as individuals who felt that PA could help them ‘get outdoors’ did 5 more occasions of PA than those who did not feel that PA could help them ‘get outdoors’. This was followed, in order, by ‘to have fun’ and ‘to control or lose weight’ with people who felt PA could help them have fun or control/lose weight doing 4 or 3 additional occasions of PA respectively.

Higher income earners (between £18,200 and £28,600) did about 3 more occasions of PA compared with lower income earners (<£10,400). The most important explanator of number of occasions of PA was health status as people with unfavourable health status did 7 extra occasions compared with those with favourable health status. Other people who did more occasions of PA were: part-time workers, unemployed, and people living alone (3 more occasions); as well as residents of North West, West Midlands, South-East and West (5 additional occasions), and South Central (7 additional occasions). Conversely, holders of National Vocational Qualification 1/Certificate of School Education equivalent qualification did 5 fewer occasions of PA compared with National Vocational Qualification 4-5/degree equivalent qualification holders.

### Swimming

#### Money prices

The number of occasions of swimming was negatively associated with occasion price for entrance to swimming facilities and monthly price for joining swimming facilities (Table 3). The former price had a greater magnitude of association with demand as a 10% higher price per entrance was associated with 29% fewer occasions of swimming, while a 10% higher monthly price for joining swimming facilities was correlated with 1% fewer occasions of swimming. Demand for swimming was positively related to monthly prices for membership (PED = 0.334), entrance (PED = 2.840), equipment (PED = 0.024), and accommodation (PED = 0.021).

#### Travel time price

Demand for swimming was travel time price inelastic as a 10% higher travel time price was associated with 5% fewer occasions on which swimming was undertaken (Table 3).

#### Control variables

Individuals who drink alcohol once every couple of months did 1 occasion less of swimming compared with people who drink alcohol almost every day. Similarly, having child/two children in the household or residing in a town was associated with about 1 occasion less of swimming. Income had a mixed relationship with number of occasions of swimming as the highest earning individuals (£46,800 and above), and those earning between £18,200 and £28,600 annually were associated with more occasions of swimming compared with people who earn less than £10,400 although the difference in demand was not statistically significant. On the other hand, earners of £10,400 - £18,200 or £28,600 - £46,800 annually did fewer occasions of swimming compared with people who earn less than £10,400. The difference in demand here was approximately 1 occasion and only statistically significant for the £10,400 - £18,200 earning group.

### Working out (using an exercise machine or weight training, either in a gym or at home)

#### Money prices

Table 3 shows that demand for workout was negatively correlated with occasion prices for entrance (PED = −0.285), classes (PED = −0.059) and monthly prices to join workout centres (PED = −0.083) and repair equipment/apparel (PED = −0.080). A positive correlation was observed for monthly prices for purchase/hiring of clothing/shoes (PED = 0.080), and family member/child care (PED = 0.024) as well as occasions prices for food/drinks (PED = 0.049), and nutritional supplements (PED = 0.035).

#### Travel time price

The relationship between travel time price and the number of occasions on which workout was undertaken is negative (PED = −0.361).

#### Control variables

Being older (aged above 25 years) was related with between 2 and 5 fewer occasions of workout, while unemployed people did an occasion less. Those with access to a vehicle undertook workout on 2 more occasions.

### Walking

#### Money prices

Demand for walking was money price inelastic (Table 3). Occasion price for walking parks was negatively related with demand (PED = −0.190) whilst monthly class fees had a positive relationship (PED = 0.305).

#### Control variables

Table 3 indicates that compared with degree level holders, people with National Vocational Qualification 1/Certificate School Education qualification walked less (8 occasions fewer). Having access to a vehicle was associated with 7 extra occasions of walking.

## Discussion

Our study shows that both high travel time and money prices per occasion of PA are associated with lower participation in PA. Family member/child care fees, parking fees, facility charges, and travel time were the key drivers of such association. Interestingly, family/child care and parking fees were correlates only in terms of demand for PA whilst court/pitch hire was associated only with specific activities. In addition, while demand for PA (in general) was travel time price elastic, the demand for specific activities was travel time price inelastic but money price elastic (entrance charge). A number of differences existed across activity specific models too. First, only demand for swimming and workout were sensitive to travel time price, entrance charge, and purchase/hiring of equipment whereas demand for walking was associated with fees for walking parks, and classes. Second, whilst for entrance charges, demand for swimming was money price elastic it was inelastic for workout. These findings not only support the importance of accounting for prices in estimating demand for PA but also suggest that a ‘generalist approach’ (e.g. free parking fees for all types of activities) may not apply to specific activities (e.g. swimming).

Further exploratory stratified analysis based on income, showed money price elasticity of demand for PA varied by income groups, with high income earners being relatively less responsive to variations in prices. For example, whilst individuals with annual household income between £10-18 k were highly sensitive to price (facility charges; PED = −4.824), those with annual household income between £18-28 k were less sensitive (parking fees; PED = −0.169).

Whilst the findings on travel time price supported the predictions of the economic theory underlying the empirical research, those on money prices were mixed in their support. The positive relationship between monthly price and demand is unsurprising because a flat monthly fee did not vary by participation in PA during the reference period of the survey (i.e. last 4 weeks) as people are likely to base their purchases on rational expectations about consumption and hence would exercise more if they incur a higher monthly price.

The interesting results were that a number of occasion prices (e.g. classes fees, and accommodation charges) were positively associated with demand whilst monthly price for facility charge, for example, was negatively related. A plausible explanation is that there are some unobservable variables that explain the positive relationship between occasion prices and demand. For example, people advised by their GP’s to do more exercise in order to alleviate or prevent a health condition are likely to do so irrespective of price. When this possibility was explored using proxy measures for exercise referral [27], respondents likely to have been referred to do more exercise paid significantly higher for the occasion prices in question. Whilst one could argue, however, that this confounding effect was captured in the model via ‘health status’, a control variable, that might not be the case because ‘health status’ was collected via HSE 2008 and may thus not fully reflect the current condition of respondents. For monthly prices, DellaVigna and Malmeinder [28] found in a study of members of three US health clubs that people tend ‘to pay not to go to the gym’ due to projection bias (overestimation of our future participation rates) or lack of self-control.

The models had good statistical properties and findings were generally consistent with a priori expectations with respect to the control variables. Comparing the results on price to the few studies that had explored the effects of price on PA unsurprisingly reveals slight differences particularly with the studies that used proxy measures for travel time price [18,22,23]. Differences in measurement of price between these studies and ours could explain why the two sets of findings vary.

A number of caveats in this study, however, justify some caution in interpretation of the findings. Although we have followed common practice in estimations of price elasticity with cross-sectional data [29,30], time-series data would be more useful. Using cross-sectional data could lead to higher price elasticities because a cross-sectional analysis assumes consumers have already responded to changes in prices and are at their long term equilibrium [31]. It is also possible that temporal trends in covariates (e.g. income, age) that might impact on price elasticity of demand are underrepresented in cross-sectional data. In addition, the variation in prices may have just been due to differences between consumers (variations in marginal cost/benefits of information search for prices) rather than supply conditions [32]. If unaccounted for, as in this study, such price variations could lead to bias and misleading elasticities [33].

A second set of issues relate to the measurement of variables. First, whilst the current specification of price (i.e. average price paid for participation) is consistent with the literature it may not be the price perceived by individuals. This might be problematic particularly in the context of PA as evidence suggests that people may base their PA behaviour on perceptions/attitudes to price and not the actual price paid per se [19,34]. Second, using self-reports to measure PA can be fraught with inaccuracies resulting from overestimation or problems with recall and these might have led to biased demand estimates. Further exploration was therefore undertaken to adjust for this measurement error (predicted probabilities for accuracy via HSE 2008) in the analysis and examine its impact on the correlates of demand. Whilst acknowledging the limitations around the timing of data availability and out-of-sample predictions, the impact of measurement error usually associated with self-report of PA could be argued to be minimal in this study as the findings were not sensitive to the adjustment of this error.

The findings indicate the potential for generating policy relevant information on demand-side incentives and suggest potential target variables that could inform the design of policies and interventions to improve PA participation. Money and travel time prices are important target variables for financial incentives aimed at subsidising participation in PA. The money price elasticity of demand suggests that any price reduction (in facility charge, family member/child care fees, and parking fees) for overall PA would need to be large to be associated with high participation in PA. However, for activities such as swimming a small price reduction could be related to a large improvement in participation for PA. For example, consider two money price reduction policies aimed at encouraging the current number of occasions on which PA is undertaken: policy ‘A’ aims at a 50% reduction in price for all PA (e.g. facility charges) and policy ‘B’, a 50% reduction in price of swimming (e.g. entrance charges). Note however, that these are two different levels of subsidies as 50% of entrance charge for swimming is not the same as 50% of facility charges for PA. Results from the model suggest that whilst the 50% lower price is associated with people doing less than an additional half an occasion of PA, it is related with two extra occasions of swimming. Thus, all things being equal, financial incentives to promote PA that target specific activities is likely to be effective. When all PA activities are targeted the effect is small. The opposite, however, occurs for policies aimed at reducing travel time price because demand for overall PA was travel time price elastic and demand for specific activities price inelastic. These observations may have been the result of the fact that the variation in both overall demand and overall average prices while combining all types of PA is much larger than that observed in a particular sport, e.g. swimming, and therefore the effect size for overall PA is underestimated. For travel time price, a plausible explanation is that the sample considered PA to be a relatively more time expensive commodity. In our sample, the proportion of time committed to PA (average of 1 hour 12 minutes per day) was higher than that of the specific activities (average of 30 minutes per day). Committing to PA (i.e. more than one activity) means it would require more time than committing to a single activity. Therefore, one would expect that individuals are more time price sensitive to PA than to a single activity.

An important consideration is the implication of our findings with respect to the debate on whether nudging and/or ‘nannyism’ is the most effective public health strategy [35,36]. Current policy/debates particularly in countries such as UK, US, Denmark, and Australia are inclined towards the former [37]. Whilst there is evidence that nudging could work with success stories emerging in the US and Denmark, for example, the findings usually come from small scale studies [38]. Our results do suggest, however, that in complex lifestyles such as physical activity, ‘nudging’ alone might not be effective [39] partly because unlike other lifestyles (e.g. smoking), in order to exercise people could be faced with a variety of money prices and time price. These prices introduce time and money constraints on individual choices that might require financial incentives to manage at least in the short-term [40].

## Conclusion

This study provides a first estimate of how participation PA (in general) and specific activities are correlated with time and money prices of participation in a general population. Findings suggest that both high travel time and money prices per occasion of PA are associated with lower participation in PA. Positive financial incentives, e.g. subsidising price of participation, could lead to an increase in quantity of PA particularly among those already exercising. However, it would be important to get more robust evidence (i.e. data over time) and from a broader range of people (e.g. those who don’t maintain exercise over time or who do not exercise per se) in order to understand these relationships better and provide conclusions that can be rooted in an entire population.

## Abbreviations

PA: Physical activity
PED: Price elasticity of demand
UK: United Kingdom
US: United States of America
